# Efflux pump genes of the resistance-nodulation-division family in *Burkholderia cenocepacia *genome

**DOI:** 10.1186/1471-2180-6-66

**Published:** 2006-07-20

**Authors:** Paola Guglierame, Maria Rosalia Pasca, Edda De Rossi, Silvia Buroni, Patrizio Arrigo, Giulia Manina, Giovanna Riccardi

**Affiliations:** 1 Dipartimento di Genetica e Microbiologia, Università degli Studi di Pavia, Via Ferrata 1, 27100 Pavia, Italy; 2 Consiglio Nazionale delle Ricerche, Istituto per lo Studio delle Macromolecole, Via De Marini 6, 16149 Genova, Italy

## Abstract

**Background:**

*Burkholderia cenocepacia *is recognized as opportunistic pathogen that can cause lung infections in cystic fibrosis patients. A hallmark of *B. cenocepacia *infections is the inability to eradicate the organism because of multiple intrinsic antibiotic resistance. As Resistance-Nodulation-Division (RND) efflux systems are responsible for much of the intrinsic multidrug resistance in Gram-negative bacteria, this study aims to identify RND genes in the *B. cenocepacia *genome and start to investigate their involvement into antimicrobial resistance.

**Results:**

Genome analysis and homology searches revealed 14 open reading frames encoding putative drug efflux pumps belonging to RND family in *B. cenocepacia *J2315 strain.

By reverse transcription (RT)-PCR analysis, it was found that *orf3*, *orf9, orf11*, and *orf13 *were expressed at detectable levels, while *orf10 *appeared to be weakly expressed in *B. cenocepacia*. Futhermore, *orf3 *was strongly induced by chloramphenicol. The *orf2 *conferred resistance to fluoroquinolones, tetraphenylphosphonium, streptomycin, and ethidium bromide when cloned and expressed in *Escherichia coli *KAM3, a strain lacking the multidrug efflux pump AcrAB. The *orf2*-overexpressing *E. coli *also accumulate low concentrations of ethidium bromide, which was restored to wild type level in the presence of CCCP, an energy uncoupler altering the energy of the drug efflux pump.

**Conclusion:**

The 14 RND pumps gene we have identified in the genome of *B. cenocepacia *suggest that active efflux could be a major mechanism underlying antimicrobial resistance in this microorganism. We have characterized the ORF2 pump, one of these 14 potential RND efflux systems. Its overexpression in *E. coli *conferred resistance to several antibiotics and to ethidium bromide but it remains to be determined if this pump play a significant role in the antimicrobial intrinsic resistance of *B. cenocepacia*. The characterization of antibiotic efflux pumps in *B. cenocepacia *is an obligatory step prior to the design of specific, potent bacterial inhibitors for the improved control of infectious diseases. Consequently, the topic deserves to be further investigated and future studies will involve systematic investigation on the function and expression of each of the RND efflux pump homologs.

## Background

Bacteria have evolved a variety of strategies to resist antibiotics such as enzymatic inactivation, target alteration, efflux, and permeability changes. Antibiotic resistance can be intrinsic or acquired. Intrinsic resistance is intimately associated to the biology of the organism and usually involves the ability to resist a large number of different classes of antibiotics, while acquired resistance occurs when a bacterium that is sensitive to antibiotics develops resistance by mutation or by acquisition of new DNA. Bacterial intrinsic drug resistance was thought to be a passive mechanism, based on the absence of the drug target or on the lack of permeability of the bacteria to a given drug. However, it is becoming increasingly evident that the intrinsic resistance of many bacteria to antibiotics depends on the constitutive or inducible expression of active efflux systems [1,2]. A typical example is *Pseudomonas aeruginosa*, which was long thought to be poorly susceptible to a large range of antibiotics of different classes due to the low level of permeability of its outer membrane to drugs. However, disruption of the gene encoding the MexB pump dramatically increases the susceptibility of *P. aeruginosa *to beta-lactams, tetracyclines, fluoroquinolones, and chloramphenicol [3], indicating that the resistance was mediated by efflux.

In the prokaryotic kingdom there are five major families of efflux transporters: MF (major facilitator), MATE (multidrug and toxic efflux), RND (resistance-nodulation-division), SMR (small multidrug resistance), and ABC (ATP binding cassette). All these systems utilize the proton motive force as an energy source, apart from the ABC family, which utilizes ATP hydrolysis to drive the export of substrates [4].

RND transporters exist in all kingdoms of living organisms, but seem to be involved in drug resistance especially in Gram-negative bacteria [5]. The RND transporters function as a protein complex spanning from the cytoplasmic membrane to the outer membrane. The recent resolution of the three-dimensional structures of TolC and AcrB from *Escherichia coli *and MexA and OprM from *P. aeruginosa *gave rise to a better understanding of the efflux mechanism in Gram-negative bacteria [6-10].

The efflux pump systems of the RND family are organized as tripartite efflux pumps. The pump in *E. coli *and other gram-negative bacteria has three components: a transporter (efflux) protein in the inner membrane (e.g., AcrB), a periplasmic accessory protein (e.g., AcrA), and an outer membrane protein (OMP) channel (e.g., TolC) [11]. AcrB captures its substrates within either the phospholipid bilayer of the inner membrane of the bacterial cell envelope or the cytoplasm [12] and transports them into the external medium via TolC [13]. The cooperation between AcrB and TolC is mediated by the periplasmic protein AcrA. The genetic organizations of the genes encoding these tripartite efflux systems are also similar among different species. Typically, the genes are organized as an operon: the regulator gene is located adjacent to the gene encoding the periplasmic accessory protein, which is located adjacent to the gene encoding the efflux pump protein, which is located next to that for the OMP. The membrane fusion protein and the pump protein are usually cotranscribed. For some systems and/or species, the OMP is not collocated with the other genes, e.g., *E. coli acrAB *and *tolC *[14] and *P. aeruginosa mexXY *and *oprM *[15].

RND efflux systems are also found in bacteria that exhibit cell wall properties to *P. aeruginosa *[16]. The *Burkholderia cepacia *complex (Bcc) is noted for their ability to cause opportunistic infections in humans, particularly in patients with cystic fibrosis (CF) [17]. Bcc infections of CF lung can result in asymptomatic carriage, chronic infection or "cepacia syndrome", which is characterized by a rapid decline in lung function that can include invasive disease [18]. The Bcc contains at least nine closely related species [19,20], of which *Burkholderia cenocepacia *(originally *B. cepacia *genomovar III) is the most prevalent and has been most commonly associated with epidemic spread and increased clinical virulence [20,21].

A hallmark of Bcc infections is the inability to eradicate the organism because of high-level multiple intrinsic antibiotic resistance [22].

The multiple antibiotic resistance of Bcc has been attributed to reduced outer membrane permeability [23-25], production of modifying enzymes such as β-lactamases [26], and alteration of antibiotic targets [27]. Much less information is available on drug efflux systems, and only a few multi-drug efflux pumps of the MFS and MATE families have been described in Bcc species [28,29]. Screening for chloramphenicol resistance of a cosmid library constructed from a CF clinical isolate allowed the identification of the first RND efflux operon in *B. cenocepacia *[30]. Within this operon, genes encoding a periplasmic lipase-like protein (*llpE*), a periplasmic linker (*ceoA*), a cytoplasmic membrane component (*ceoB*), and an outer membrane protein (*opcM*) were identified [31]. CeoA, CeoB, and OpcM have homologs in *P. aeruginosa *and other Gram-negative bacteria. A significant difference from other prokaryotic multidrug efflux systems, including all RND pumps described to date, is the inclusion of a gene encoding a lipase-like protein in the *B. cenocepacia *efflux operon [31].

Aim of this study was to identify RND genes in the *B. cenocepacia *genome and start to investigate their involvement into antimicrobial resistance. Fourteen homologues of RND efflux pump genes were identified by *in silico *homology searches. By RT-PCR it was found that some of them were expressed at detectable levels in not inducing condition, i.e. growth in LB medium, while others were detected in inducing conditions, i.e. growth in LB medium in the presence of an antibiotic. Further, *orf2 *gene conferred a multidrug resistant phenotype, when over-expressed in *Escherichia coli *KAM3 strain. Moreover, an energy-dependent ethidium bromide efflux was observed in *E. coli *KAM3 cells harbouring *orf2 *gene.

## Results and discussion

### Analysis of RND drug transporters

The *B. cenocepacia *J2315 strain was isolated in 1989 in Edimburgh (UK) from the sputum of a cystic fibrosis patient [[32], in this reference identified as CF5610]. The genome sequence of this strain is available within the Sanger website [33]. To identify the *B. cenocepacia *drug efflux proteins belonging to RND, we first scanned the *B. cenocepacia *predicted proteins for the presence of the transporter family signature characterized by a number of strongly conserved amino acid residues. The sequences of the motifs are: motif A (G x s x v T v x F x x g t D x x x A q v q V q n k L q x A x p x L P x x V q x q g x x v x k), motif B (a l v l s a V F l P m a f f g G x t G x i y r q f s i T x v s A m a l S v x v a l t l t P A l c A), motif C (x x x G k x l x e A x x x a a x x R L R P l L M T s L a f i l G v l P l a i a t G x A G a), and motif D (S i N t l T l f g l v l a i G L l v D D A l V v V E N v e R v l a e) [34], where x indicate any amino acid, capital letters show amino acids most frequently observed in a single position in more than 70% of the transport proteins, and lowercase letters display amino acid occurring in more than 40% of RND. By this method, fourteen possible RND homologues have been identified (here named ORF1-ORF14). In Table 1, the position of the residues encompassing the four typical motifs is indicated; it is noteworthy that the relative distances among the specific motifs appear well conserved in all the identified proteins.

Members of the RND family have 12 TMS, with two large loops between TMS1 and TMS2, and TMS7 and TMS8, respectively [5]. This organization appears well conserved in all the *B. cenocepacia *hypothetical RND transporters, except for ORFs 11 and 14, which present 11 and 10 TMS, respectively (data not shown). In order to assess a putative function of the identified transporters, an integrative analysis of protein motif databases was carried out, by using the InterProScan program. The proteins ORF1–ORF10 show the ACRIFLAVINRP, a 9-element fingerprint that provides a signature for members of the acriflavin resistance protein family as AcrB of *E. coli *[35], while the proteins ORF11–ORF14 show a 6–8 element fingerprint (Table 1). All hypothetical RNDs contain two transmembrane functional domains located within TMS4 and TMS11 that coincide with the transporter family signature motifs C and D, respectively (Table 1). The crystal structure of multidrug efflux transporter AcrB of *E. coli *[10] reveals that this protein is characterized by the four subdomains PN1, PN2, PC1, and PC2. The structural motifs PN1 and PN2 comprise the polypeptide segment between TMS1 and TMS2, while PC1 and PC2 comprise the segment between TMS7 and TMS8. All the *B. cenocepacia *RND transporters (ORF1–ORF14) show the four subdomains; the region with subdomains PN1 and PN2 coincides always with the transporter family signature motif A (Table 1). The TolC docking domain is composed of two subdomains, DN and DC, and these subdomains are found in all hypothetical RND proteins except the ORF11 that present only the DC subdomain (Table 1). In order to obtain a more exhaustive analysis about the structural and conformational properties of the ORFs, the amino acid sequences were analysed by Phyre program and the data were reported in Table 1. The ORF1–14 were compared to the RND proteins of *E. coli *and *P. aeruginosa *by using the BLASTP program. Particularly, ORF1, ORF2, and ORF4 show strong identity with *E. coli *AcrB (60%, 64%, 66%, respectively) and with *P. aeruginosa *MexB (55%, 63%, 64%, respectively). ORF5 and ORF8 show identity with *P. aeruginosa *MexD (50%, 56%, respectively), while ORF9 and ORF10 show identity with *P. aeruginosa *MexF (56%, 64%, respectively). ORF11 and ORF12 are homologues of metal efflux transporters belonging to the RND family of *E. coli *and *P. aeruginosa *(data not shown). Particularly, ORF11 is highly homologous to CzrA protein of *P. aeruginosa*, involved in cadmium and zinc resistance [36].

**Figure 1:**
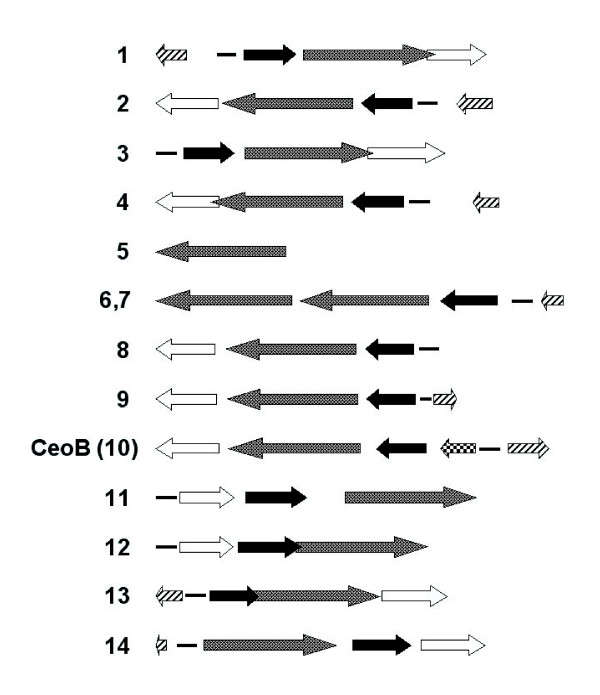
**Proposed RND efflux pump encoded by *B. cenocepacia *genome**. The organization of the RND efflux pump genes identified in *B. cenocepacia *genome was achieved by bioinformatic tools as described in Methods. RND transporter-encoding genes are depicted as grey arrows (*orf1–orf14*), outer-membrane factor-encoding genes as white arrows, periplasmic membrane fusion protein-encoding genes as black arrows, and regulatory genes as arrows with stripes. Black lines indicate the regions of Stress-Induced DNA duplex Destabilization that could be associated with promoters. Genes encoding protein components of CeoAB-OpcM (6) are indicated as white arrow (*opcM*), grey arrow (*ceoB*), black arrow (*ceoA*); arrows with stripes and squares indicated the *ceoR *and *llpE *genes encoding a regulator and a putative periplasmic lipase-like protein, respectively.

Analysis of the upstream and downstream regions of the RND coding genes confirmed the association with both an AcrA/MexA homolog and a TolC/OprM homolog in a variety of arrangements (Figure 1). Indeed, *orf1, orf2, orf3, orf4, orf8, orf9, orf10 *(*ceoB*), *orf11, orf12, orf13*, and *orf14 *seem to be part of an operon that also included a *mexA *and an *oprM *homologs, thus containing all three of the components of the tripartite pump complex. Differently, *orf5 *appears to have only the membrane component, while *orf6 *and *orf7 *are apparently co-transcribed and in operon with a periplasmic membrane fusion protein (Figure 1). In *P. aeruginosa*, the genes encoding the RND transporter (MexB homolog) and periplasmic membrane fusion protein (MexA homolog) are always present, while the gene encoding the OM channel (OprM homolog) is not always present [37]. In *E. coli*, the genes for transporter and membrane fusion proteins (*acrAB*) occur together as an operon and the gene for the TolC occurs elsewhere on the genome [37]. In *B. cenopacia *the genetic organization of the RND *orfs *seems to resemble in part that of *P. aeruginosa *and in part that of *E. coli*. As AcrD pump of *E. coli*, *orf5 *seem to be transcribed alone in the genome of *B. cenocepacia *[38], while *orf6 *and *orf7 *are organized as the genes *PA2526*-*2527*-*2528 *of *P. aeruginosa *that contains an additional RND transporter [39]. Many pump component-encoding operons contain a physically linked regulatory gene coding for either a repressor or an activator [40]. For example, expression of the *mexAB-oprM *operon is regulated by the product of the upstream and divergently transcribed *mexR *gene. Not surprisingly, clinical isolates that overexpress the MexAB-OprM system often carry mutations in the *mexR *gene [41]. In the case of *orf1*, *orf2, orf4, orf6–7*, *orf9, orf10*, *orf13*, and *orf14 *a hypothetical regulatory gene has been identified, divergently transcribed for all genes, except for the hypothetical operon containing the *orf2*, *orf4*, and *orf6–7*, respectively (Figure 1). The WEBSIDD program was used to analyse sequences encompassing the coding and intergenic regions of the hypothetical RND operons for the presence of SIDD (Stress Induced Duplex Destabilization) sites. These sites have a specific and statistically highly significant pattern of association with transcriptional regions, specifically with promoters because strand separation is a necessary step in the initiation of transcription [42]. All the ORFs present SIDD sites localized upstream of the first gene of the entire operon, except for the *orf5*.

### RT-PCR analysis of the *B. cenocepacia *genes coding for the RND transporters

**Figure 2:**
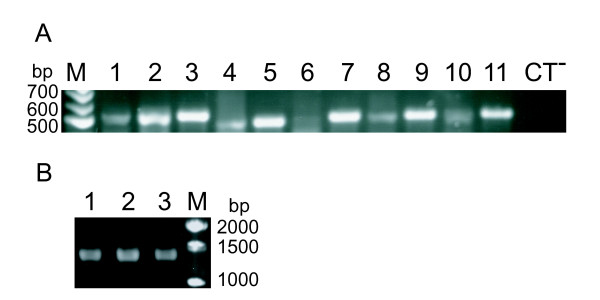
**Expression of genes coding for the RND proteins from *B. cenocepacia***. Expression of genes coding for the RND proteins was evaluated by RT-PCR of RNA isolated from *B. cenocepacia *cultures grown in the LB standard medium without and with chloramphenicol. A) Lane M represents the molecular weight marker (Fermentas), whose sizes of some bands in base pairs (bp) are provided on the left; lanes 3, 5, 7, 9, and 11 represent positive controls, i.e. genomic DNA amplification with primers specific for *orf3*, *orf9*, *orf10*, *orf11*, and *orf13 *(Table 3); lanes 1 and 2 represent RT-PCR amplification of *orf3 *in the absence and in the presence of 12.8 μg/ml (0.4X MIC) of chloramphenicol, respectively; lanes 4, 6, 8, and 10 represent RT-PCR amplifications of *orf9*, *orf10*, *orf11*, and *orf13*, without antibiotic, respectively; lane CT represents the PCR reaction negative control. B) Lanes 1 and 2 represent the expression of *rRNA *16S gene in the presence or in the absence of chloramphenicol, respectively; lane 3 represents the positive control, i.e. genomic DNA amplification with primers specific for *rRNA *16S gene; lane M represents the molecular weight marker (Fermentas), whose sizes of some bands in base pairs (bp) are provided on the right.

We found 14 genes codifying hypothetical RND drug transporters into *B. cenocepacia *genome. By RT-PCR analysis, we studied the expression of all these genes at the exponential phase of cells grown in LB medium. A clear expression of *orf3*, *orf9*, *orf11*, and *orf13 *was observed (Figure 2A, lanes 1, 4, 8,10), while *orf10 *appeared to be weakly expressed (Figure 2A, lane 6). The expression of the other *orfs *(*orf1, orf2, orf4, orf5, orf6–7, orf8, orf12*, and *orf14*) was not detectable, at least in our conditions (data not shown).

It has been demonstrated that *orf10 *corresponds to the previously described *ceoB *gene belonging to *ceo *operon, which has been shown to be strongly induced by salicylate and chloramphenicol [31]. We decide to evaluated the expression of the 14 genes in *B. cenocepacia *cells grown in the presence of chloramphenicol (inducing conditions). In *B. cenocepacia *J2315 strain, the basal level of expression of the *orf3 *(Figure 2A, lane 1) was strongly increased by chloramphenicol (Figure 2A, lane 2). No induction could be detected in the case of *orf1, orf2, orf4, orf5, orf6–7, orf8, orf12*, and *orf14 *(data not shown) and no increased expression could be observed in the case of *orf9*, *orf10*, *orf11*, and *orf13 *(data not shown). However, the *B. cenocepacia rRNA *16S expression was not affected by different growth conditions (Figure 2B, lanes 1 and 2), thus demonstrating that the differences detected in the amount of *orf3 *mRNA, under different growth conditions, are genuine.

As the *ceo *operon has been discovered as responsible for the MDR phenotype of a clinical strain [31], we can hypothesize that this RND efflux system is expressed as a result of a mutation. In our conditions, no transcriptional induction of *orf10 *could be detected also in the presence of salicylate (data not shown).

ORF3 showed 71% identity with *P. aeruginosa *MexY, an inducible efflux system that contributes to the natural resistance of *P. aeruginosa *to antibiotics [43]. Experiments involving real-time PCR in *P. aeruginosa *PAO1 showed induction of gene *mexY *by chloramphenicol, tetracycline, macrolides, and aminoglycosides [44]. To date, no information is available on the regulation of *mexXY *expression, except that mutations occurring in the divergently transcribed repressor gene *mexZ *are frequently isolated from the sputa of cystic fibrosis patients [45]. In *P. aeruginosa*, with the exception of MexAB-OprM, the expression of most of the RND efflux systems is tightly regulated and overexpression of these RND pumps is usually caused by mutations in genes encoding regulatory proteins [39]. Notably, overexpression of multidrug resistance pumps in clinical isolates of *P. aeruginosa*, resulting in increased bacterial resistance, is usually due to mutations in these regulatory genes [46,47]. It would be interesting to further investigate if the expression of *orf3*, *orf9*, *orf11*, and *orf13 *is due to a constitutive expression or to a transcriptional deregulation in the *B. cenocepacia *clinical isolate analysed in this study, which showed a multidrug resistance phenotype as it is resistant to ampicillin and aztreonam (>2048 μg/ml), ceftazidime (768 μg/ml), meropenem (64 μg/ml), piperacillin (768 μg/ml) amikacin (64 μg/ml), norfloxacin (32 μg/ml), rifampicin (256 μg/ml), streptomycin (>2048 μg/ml), tetracycline (128 μg/ml), chloramphenicol (32 μg/ml), and gentamicin (1536 μg/ml).

### Cloning and phenotypic analysis of *B. cenocepacia orf2 *into *E. coli *KAM3

One approach to test the role of the identified genes in drug efflux is to clone them into suitable hosts by selecting on different compounds. In spite of several attempts, we met many problems in cloning experiments since a lot of rearranged clones were found in the host strains (*E. coli *and *B. multivorans*). The difficulties that we met in cloning experiments could rely both on plasmid instability and/or toxic effect of the gene product (data not shown). In fact, the failure could be due to the toxicity of the gene product for the host bacterial cells and still *B. multivorans *as a host causes many troubles (i.e. recombinations, overlapping with existing pumps, etc.).

To overcome this problem we used the *E. coli *KAM3 strain as host, which lacks the *acrAB *genes and is sensitive to many drugs that are known as substrates of the AcrAB system [48]. We decided to characterize one of the 14 hypothetical drug efflux pumps of *B. cenocepacia *by cloning the *orf2*-coding region from *B. cenocepacia *genomic DNA into pBAD202 expression vector. The pBAD202 vector and the recombinant plasmid (pBAD/*orf2*) were transformed into *E. coli *KAM3. We chose ORF2 because of its high homology to MexB, responsible for antibiotic resistance in many *Pseudomonas *clinical isolates [3]. Particularly, studies with mutants that overproduce or lack MexAB-OprM demonstrated that this efflux system extrudes quinolones, macrolides, tetracycline, chloramphenicol, novobiocin, and most β-lactams but not imipenem [3,49].

The MICs of *E. coli *KAM3 strains harbouring pBAD202 and pBAD/*orf2*, respectively, were determined by streaking the cultures onto LB containing different concentrations of the following compounds: streptomycin, ethidium bromide, nalidixic acid, several quinolones (ciprofloxacin, ofloxacin, norfloxacin, and sparfloxacin), chloramphenicol, erythromycin, tetraphenylphosphonium, and tetracycline. Plates were incubated at 37°C for 3 days and the growth was visually evaluated. Compared to the control strain, the overexpression of *orf2 *into *E. coli *KAM3 cells conferred resistance to streptomycin (16X MIC), tetraphenylphosphonium (8X MIC), ethidium bromide (4X MIC), nalidixic acid (4X MIC), ciprofloxacin (4X MIC), ofloxacin (2X MIC), and norfloxacin (2X MIC) (Table 2). No difference in drug susceptibility was found with sparfloxacin, chloramphenicol, erythromycin, and tetracycline (data not shown). These data suggest that *orf2 *is expressed and functional in *E. coli *KAM3. Interestingly, in *P. aeruginosa*, it has been found that fluoroquinolones like nalidixic acid and norfloxacin can commonly select mutants that constitutively overproduce the MexAB-OprM efflux pump system [46].

From these data it appeared that streptomycin and norfloxacin could be substrates of ORF2. We decided to perform RT-PCR of *B. cenocepacia *grown in the presence of sub-inhibitory concentration of streptomycin (204.8 μg/ml) and norfloxacin (3.2 μg/ml). In our condition, *orf2 *transcript could not be detected by RT-PCR (data not shown). The expression of *orf2 *was also evaluated by Southern blotting of the RT-PCR agarose gel and subsequent hybridization with a labelled 511-bp fragment internal to *orf2 *coding region. No hybridisation signals were observed (data not shown), indicating that *orf2 *is not expressed following treatment of *B. cenocepacia *cells with streptomycin or norfloxacin.

Three different hypotheses could be given to explain the fact that *orf2 *is not expressed. First, the experimental approaches utilized in this work (RT-PCR and Southern hybridization analysis) are not enough sensitive to detect very small amount of *orf2 *mRNA. Real-time PCR is an attractive method for estimating gene expression of efflux pumps in bacteria because of its great sensitivity and wide effective range. Recently, the real-time PCR appeared to be a useful alternative method for assessing the expression of efflux pumps MexB and MexY in *P. aeruginosa *[50]. Second, not all ORF2 substrates in *E. coli *necessarily must be inducers of the expression of this pump in *B. cenocepacia*. A relevant example of such a behaviour is given by the MexCD-OprJ efflux pump of *P. aeruginosa*, which extrudes antibiotics that do not induce its expression [51]. Instead, expression of this efflux system was induced by clinical important disinfectants such as benzalkonium chloride and chlorhexidine gluconate [51]. Finally, the *orf2 *expression may occur in a phase of cellular growth different from the exponential one we tested. In conclusion, we could not determined neither the real inducing agent nor the culture conditions in which induction of *orf2 *expression is triggered; therefore, it could be interesting to test other organic compounds for the ability to induce expression of this efflux pump.

### Ethidium bromide efflux activity

Ethidium bromide efflux experiment was performed to determine whether *E. coli *KAM3 cells, carrying the recombinant plasmid pBAD/*orf2*, were more resistant to this dye due to an active efflux mechanism.

**Figure 3:**
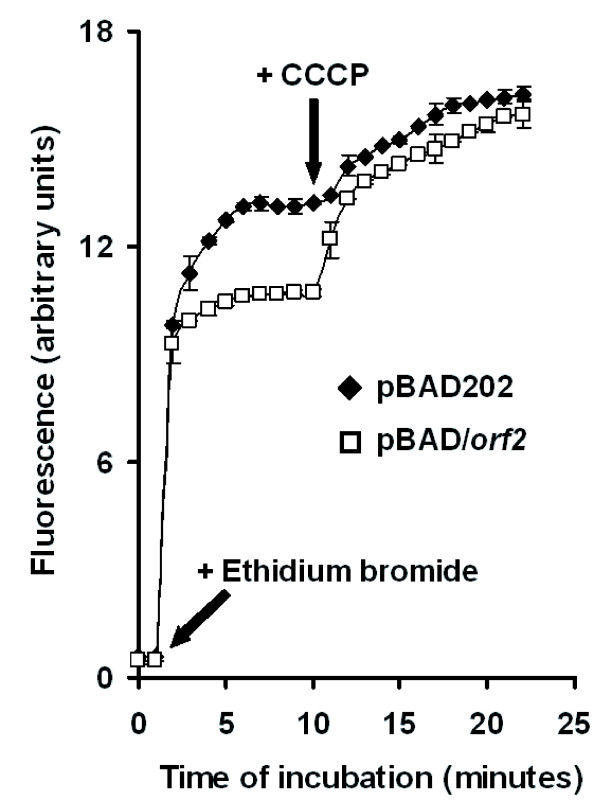
**Accumulation of ethidium bromide in *E. coli *KAM3 cells harbouring pBAD202 and pBAD/*orf2***. *E. coli *KAM3 cells containing the cloning vector pBAD202 and the recombinant plasmid pBAD/*orf2 *were grown in LB medium at 37°C and treated as described in Methods. Ethidium bromide was added to cell suspensions carrying pBAD202 (◆) or pBAD/*orf2 *(□), at a final concentration of 20 μM whereas the efflux inhibitor CCCP was added 9 min after the addition of ethidium bromide at a final concentration of 30 μM. The experimental error bars showed standard deviations of triplicate determinations.

As shown in Figure 3, cells harboring the cloning vector pBAD202 take up ethidium bromide rapidly and achieve a steady-state level of accumulation within about 5 min of incubation.

As observed in several experiments, accumulation of ethidium bromide was approximately 20% lower when cells contained the pBAD/*orf2 *recombinant plasmid.

A reduced level of accumulation of the drug may be caused either by a decreased level of drug permeation or by active drug extrusion through the cytoplasmic membrane. To study the effect of membrane deenergization on the uptake of tetracycline, the protonophore carbonyl cyanide *m*-chlophenylhydrazone (CCCP) was added to cells containing ethidium bromide. Upon the addition of CCCP, the level of ethidium bromide accumulation increased in the case of the pBAD/*orf2*-harboring strain and reached a level almost equal to that observed in the case of the strain containing only the cloning vector pBAD202 (Figure 3). On the contrary, under our conditions, CCCP had no significant effect on the level of ethidium bromide accumulation in the strain carrying the cloning vector (Figure 3). These data indicate that ORF2 pumped out ethidium bromide in an energy-dependent process, presumably by using proton motive force. We could conclude that ORF2, belonging to RND transporters, is an efflux pump of *B. cenocepacia *J2315.

## Conclusion

Pulmonary infection with Bcc in patients with CF is often associated with a more rapid decline in lung function. Because of the resistance of Bcc to many antibiotics, treatment options are often limited.

The emergence of active efflux as a major causative factor in antibiotic resistance has been one of the most significant trend in anti-infective chemotherapy over the last decade and strategies to identify efflux pump inhibitors are in progress [52]. For these reasons, the identification and the characterization of such efflux systems in *B. cenocepacia *are a very important topic in the field of antibiotic resistance.

Taken together the results described in this paper fit with the prediction that *B. cenocepacia *contains homologs of the *P. aeruginosa *Mex-Opr efflux pump systems. Moreover, *orf3, orf9, orf11*, and *orf13 *are expressed in *B. cenocepacia *J2315, even if, at this stage, it is not possible to state if this expression is constitutive or due to mutations in regulatory genes.

Finally, the results described in this paper suggested that at least one of the RND-type efflux pumps identified, extrude out of the cell some antibiotics as well as other compounds (e.g. ethidium bromide). To demonstrate that this pump is really involved in the multidrug resistant phenotype of the *B. cenocepacia *J2315, further studies are necessary. The generation of mutants with pump overproduction and/or genetic deletion of genes encoding pumps in a controlled laboratory environment provides invaluable information on the potential impact of multidrug efflux pumps on the intrinsic resistance of *B. cenocepacia *J2315. Because of the difficulties in cloning and expression experiments, knock-out approach represents the only alternative method to physiologically characterize these transporters and to confirm if the encoded pumps play a significant role in the antimicrobial resistance of this microorganism.

A greater understanding of the genetic determinants that play a role in antibiotic resistance will lead to the development of new strategies for the treatment of *B. cenocepacia *infections in patients with cystic fibrosis.

## Methods

### Computer methods

The *B. cenocepacia *J2315 genome sequence data were produced by the *B. cenocepacia *Sequencing Group at the Sanger Institute and it was retrieved from the Sanger FTP Server [53]. This analysis integrates different approaches and is based on the conservation of three features among drug transporters in microorganisms: specific sequence motifs, overall sequence similarity, and structural similarities.

Searches of the stretches of amino acids containing the specific motifs conserved in members of the RND transporters among the *B. cenocepacia *proteins were done using the BLAST SEARCH program provided at the Sanger website [33]. To well characterize the specific motifs present in the *B. cenocepacia *transporters, the regions containing the motifs were aligned using the CLUSTALW program within the EBI website [54]. Searches of sequence similarity between potential drug transporters and proteins characterized in *E. coli *and *P. aeruginosa *were done using the BLASTP program within the NCBI website [55]. The number and the position of the transmembrane domains (TMS) have been predicted by TMHMM program within the CBS website [56]. All the identified hypothetical RND proteins were analysed by InterProScan program provided by the EBI website [54] and Phyre program provided by the SBG website [57]. InterPro is a database of protein families, domains, and functional sites in which identifiable features found in known proteins can be applied to unknown protein sequences; the Phyre automatic fold recognition server predicts the structure and/or function of unknown protein sequences.

The analysis for the presence of duplex destabilized motifs in sequences containing the intergenic and coding regions encompassing RND genes were done using WEBSIDD program within the Genome Center website [58]. This program uses an algorithm that allows to identify regions of Stress-Induced DNA duplex Destabilization (SIDD) that could be associated with promoters [59].

### Growth conditions, plasmids, and cloning procedures

*Escherichia coli *KAM3 and *Burkholderia cenocepacia *J2315 strains were grown on Luria Bertani agar or broth (LB) medium at 37°C. *E. coli *KAM3 cells harbouring pBAD202 and pBAD/*orf2 *plasmids were grown in the presence of kanamycin (50 μg/ml) (Sigma).

*B. cenocepacia *J2315 genomic DNA was isolated as described previously by Scordilis *et al*. [60].*orf2 *was amplified by PCR from *B. cenocepacia *J2315 genomic DNA, by using the 2pbadFOR and 2pbadREV primers indicated in Table 3. The CACC sequence was introduced at 5' terminus of the primer 2pbadFOR to directionally clone the PCR product in the correct orientation into pBAD202 expression vector (as described by "pBAD directional TOPO expression kit" of Invitrogen).

The PCR reaction was carried out as follows by using AccuPrime Pfx DNA polymerase (Invitrogen): initial denaturation at 95°C for 3 min; 35 cycles of denaturation at 95°C for 30 sec, annealing at 52°C for 1 min, extension at 68°C for 4 min and final extension at 68°C for 10 min. PCR products were analysed by electrophoresis on 1% agarose gel, containing 0.5 μg/ml of ethidium bromide and visualized under UV light.

The *orf2 *PCR product was cloned into pBAD202 expression vector and transformed into *E. coli *Top10 competent cells (Invitrogen). Plasmid DNA, isolated by the alkaline lysis method [61], was sequenced to be sure that no mutations were introduced in the amplified product and introduced into *E. coli *KAM3 strain by electroporation.

### RT-PCR

RT-PCR was used to monitor gene expression. *B. cenocepacia *J2315 strain was grown to O.D._600 _= 0.3 in LB broth. To detect if gene expression was induced by some substrates, cells were grown in the presence of chloramphenicol at a final concentration of 12.8 μg/ml (0.4X MIC), streptomycin at a final concentration of 204.8 μg/ml (0.1X MIC), and norfloxacin at a final concentration of 3.2 μg/ml (0.1X MIC). Total RNA was extracted using the RNeasy Mini Kit (Qiagen), according to the manufacturer's protocol. 1 μg of RNA was treated with 10 U of DNase I-RNase free (Roche) for 30 min at room temperature and then heated at 70°C for 10 min. The RT reactions were carried out, by using 2 μg of template RNA in the presence of M-MLV Reverse Transcriptase (Promega) and specific gene primers (Table 3). Reverse transcription was carried out as follows: (a) 2 μg of RNA and 0.5 μg of each downstream primer were incubated at 70°C for 5 min and then cooled on ice; (b) 5 μl of 5X reaction buffer, 5 μl of 10 mM dNTPs, and 200 U of M-MLV enzyme were added and the reaction was carried out at 37°C for 1 h; (c) the enzyme was inactivated at 95°C for 5 min and the reaction was ethanol-precipitated. The cDNA was dissolved in 20 μl of deionised water and 4 μl were used as template with the gene-specific primers (Table 3) for PCR amplification, by using RedTaq genomic DNA polymerase (Sigma). The PCR reaction was carried out as follows: initial denaturation at 94°C for 3 min; 25 cycles of denaturation at 94°C for 1 min, annealing at 54°C, 62°C, 64°C, 66°C or 68°C depending on the primer for 1 min, extension at 72°C for 1 min, and final extension at 72°C for 10 min. RT-PCR products were analysed by electrophoresis on 1.5% agarose gel, containing 0.5 μg/ml of ethidium bromide and visualized under UV light. The same reactions were carried out for each sample without M-MLV Reverse Transcriptase to ensure that amplification was a result of cDNA and not of contaminating DNA molecules. In all experiments, the expression of rRNA 16S gene was determined as an internal control to ensure that the differences observed in genes expression were not due to variability in the RNA isolation and/or in the RT-PCR technique.

Southern blotting was performed as described by Sambrook and Russell [61]; a 511-bp fragment internal to *orf2 *coding region was labelled with [α-^32^P] dCTP 3000 Ci mmol^-1 ^(Amersham Biosciences) by using the HexaLabel Plus DNA Labelling Kit (Fermentas) according to the manufacturer's instructions.

### MIC determinations

Determination of MIC for *E. coli *KAM3, transformed with recombinant plasmids, was performed by streaking 1 × 10^5 ^cells onto LB agar containing different concentrations of drugs. The following compounds were tested: streptomycin, ethidium bromide, nalidixic acid, several quinolones (ciprofloxacin, ofloxacin, norfloxacin, and sparfloxacin), chloramphenicol, tetraphenylphosphonium erythromycin, and tetracycline. Plates were incubated at 37°C for 3 days and the growth was visually evaluated.

Determination of MIC for *B. cenocepacia *J2315 strain was performed by streaking 1 × 10^5 ^cells onto LB containing different concentrations of drugs. Plates were incubated at 37°C for about 3 days and the growth was visually evaluated. The following antibiotics were tested to determine the resistance profile: ampicillin, amikacin, aztreonam, ceftazidime, chloramphenicol, gentamicin, meropenem, norfloxacin, piperacillin, tetracycline, streptomycin, and rifampicin.

The MIC is defined as the lowest concentration of drug that prevented visible growth. The results are the averages of three replicates.

### Ethidium bromide accumulation assay

*E. coli *KAM3 cells harbouring pBAD202 and pBAD/*orf2 *plasmids were grown in LB medium in the presence of kanamycin (50 μg/ml) under aerobic conditions at 37°C. Bacteria were harvested at the esponential phase of growth, washed twice with a minimal medium [76 mM (NH_4_)_2_SO_4_, 500 mM KH_2_PO_4_, pH 7.0, 1 mM MgSO_4_, 0.4% glucose], and resuspended in the same medium to an O.D._650 _of 0.2. Ethidium bromide was added to cells to a final concentration of 20 μM and the change in fluorescence intensity was continuously monitored at excitation and emission wavelengths of 500 and 580 nm, respectively. Fluorescence intensity is proportional to the quantity of intracellular dye, since ethidium bromide fluorescence is enhanced by the binding to intracellular components, especially to nucleic acids.

The efflux pumps inhibitor carbonyl cyanide *m*-chlophenylhydrazone (CCCP) was added to the cells suspensions to a final concentration of 30 μM after 9 min of the addition of ethidium bromide, to assess energy-dependent efflux.

## Authors' contributions

PG: carried out BLAST SEARCH, BLASTP, CLUSTALW, and TMHMM analyses, and efflux experiments. MRP: performed cloning experiments, MIC determinations, and manuscript preparation. EDR: performed experimental coordination. SB: performed RT-PCR experiments. PA: performed analysis of RND proteins by InterProScan and Phyre programs, and RND genes analysis by WEBSIDD program. GM: several cloning attempts with different vectors. GR: design of the study and coordination. All authors read and approved the final manuscript.

## Figures and Tables

**Table 1:** Predicted functional motifs for ORF1-ORF14 of *B. cenocepacia*

	**RND CONSERVED MOTIFS**	**FUNCTIONAL MOTIFS**				**STRUCTURAL ANALYSIS**
	
**ORFs^a^**	**Motif s A^b^, B^b^, C^b^, D^b^**	**Acriflavin resistance protein domains (ACRIFLAVINRP)^c^**	**AcrB transmembrane domain^c^**	**AcrB pore domain (PN1, PN2, PC1, PC2 subdomains)^c^**	**AcrB/TolC docking domain (DN, DC subdomains)^c^**	**Family^d^**
BCAS0592 **ORF1 **Chromosome 3	38–134, 445–493, 826–1051, 272–503	8–32, 36–54, 336–359, 363–384, 391–415, 445–468, 470–493, 552–569, 620–634	272–503, 826–1051	38–134, 565–669	182–271, 720–825	Multidrug efflux transporter AcrB pore domain
BCAS0765 **ORF2 **Chromosome 3	38–134, 445–493, 809–1035, 272–503	8–32, 36–54, 336–359, 363–384, 391–415, 445–468, 470–493, 553–570, 622–636	272–503, 809–1035	38–134, 566–672	182–271, 723–808	Multidrug efflux transporter AcrB transmembrane domain
BCAL1675 **ORF3 **Chromosome 1	38–133, 444–492, 804–1027, 271–502	8–32, 36–54, 335–358, 362–383, 390–414, 444–467, 469–492, 551–568, 618–632	271–502, 804–1027	38–133, 564–668	719–803, 181–270	Multidrug efflux transporter AcrB transmembrane domain
BCAL2821 **ORF4 **Chromosome 1	38–134, 445–493, 809–1035, 272–503	8–32, 36–54, 336–359, 363–384, 391–415, 445–468, 470–493, 553–570, 622–636	272–503, 809–1035	38–134, 566–672	182–271, 723–808	Multidrug efflux transporter AcrB transmembrane domain
BCAL1778 **ORF5 **Chromosome 1	40–136, 446–494, 812–1037, 274–504	10–34, 38–56, 338–361, 365–386, 393–417, 446–469, 471–494, 562–579, 629–643	274–504, 812–1037	40–136, 575–676	184–273, 727–811	Multidrug efflux transporter TolC docking domain
BCAL1079 **ORF6 **Chromosome 1	40–136, 438–486, 792–1019, 266–496	10–34, 38–56, 330–353, 357–378, 385–409, 438–461, 463–486, 543–560, 610–624	266–496, 792–1019	40–136, 556–656	189–265, 707–791	Multidrug efflux transporter AcrB pore domain
BCAL1080 **ORF7 **Chromosome 1	40–134, 438–486, 853–1084, 266–496	10–34, 38–56, 330–353, 357–378, 385–409, 438–461, 463–486, 544–561, 607–621	266–496, 853–1084	40–134, 557–653	183–265, 704–776	Multidrug efflux transporter AcrB pore domain
BCAM0926 **ORF8 **Chromosome 2	38–134, 445–493, 803–1027, 272–503	8–32, 36–54, 336–359, 363–384, 391–415, 445–468, 470–493, 552–569, 619–633	272–503, 803–1027	38–134, 565–667	182–271, 718–802	Multidrug efflux transporter AcrB pore domain
BCAM1946 **ORF9 **Chromosome 2	40–136, 447–495, 814–1041, 275–505	10–34, 38–56, 339–362, 366–387, 394–418, 447–470, 472–495, 562–579, 631–645	275–505, 814–1041	40–136, 575–678	185–274, 729–813	Multidrug efflux transporter TolC docking domain
BCAM2550 **ORF10 **Chromosome 2	40–136, 447–495, 817–1043, 275–505	10–34, 38–56, 339–362, 366–387, 394–418, 447–470, 472–495, 562–579, 631–645	275–505, 817–1043	40–136, 575–681	185–274, 732–816	Multidrug efflux transporter AcrB pore domain
BCAM0713 **ORF11 **Chromosome 2	42–135, 480–503, 822–1049, 167–513	12–36, 40–58, 339–362, 366–387, 480–503, 553–570	167–513, 822–1049	42–135, 566–673	726–821	Multidrug efflux transporter TolC docking domain
BCAM0435 **ORF12 **Chromosome 2	42–135, 454–502, 807–1043, 269–511	12–36, 40–58, 334–357, 361–382, 389–413, 552–569	269–511, 807–1043	42–135, 565–610	185–268, 722–806	Multidrug efflux transporter AcrB pore domain
BCAL1811 **ORF13 **Chromosome 1	49–146, 446–494, 805–1029, 274–504	47–65, 339–362, 393–417, 446–469, 471–494, 554–571	274–504, 805–1029	49–146, 567–677	192–273, 721–804	Multidrug efflux transporter AcrB pore domain
BCAS0582 **ORF14 **Chromosome 3	40–136, 438–486, 832–1060, 266–496	10–34, 38–56, 330–353, 357–378, 385–409, 438–461, 463–486, 551–568	266–496, 832–1060	40–136, 564–675	183–265, 727–831	Multidrug efflux transporter AcrB pore domain

^a^As reported by the web site: ; ^b^Motifs derived from the BLAST SEARCH program at the web site: ; ^c^Results from the web site: ; ^d^Results from the web site: .

**Table 2:** MIC (μg/ml) of drugs for *E. coli *KAM3 cells transformed with pBAD202 and pBAD/*orf2 *plasmids

	**pBAD202**	**pBAD/*orf2***
**Streptomycin**	0.5	8 (16X)
**Tetraphenylphosphonium**	4	32 (8X)
**Ethidium bromide**	8	32 (4X)
**Nalidixic acid**	0.25	1 (4X)
**Norfloxacin**	0.0125	0.025 (2X)
**Ofloxacin**	0.002	0.004 (2X)
**Ciprofloxacin**	0.001	0.004 (4X)

**Table 3:** Primers used in this study

**Primer**	**Sequence of primers**	**Comments**	**Product size (bp)**
**2pbadFOR**	5'-CACCATGGCCAAGTTCTTTATC-3'	Sense primer for the cloning of *orf2*-coding into pBAD202	3192
**2pbadREV**	5'-TTACTGCCCCTTCGAATCGTTT-3'	Antiense primer for the cloning of *orf2*-coding into pBAD202	
**1rtFOR**	5'-CCACGCCGTTCGTTCGCATCT-3'	Sense primer for RT-PCR analysis of *orf1*	577
**1rtREV**	5'-GCTTGGCGAACCACCGGTTGA-3'	Antisense primer for RT-PCR analysis *orf1*	
**2rtFOR**	5'-CGAAGCTGAAGCCGTTGAC-3'	Sense primer for RT-PCR analysis *orf2*	511
**2rtREV**	5'-TCGTGCTGTCGGCGGTGTT-3'	Antisense primer for RT-PCR analysis *orf2*	
**3rtFOR**	5'-CGCGCTCACCGACCTGATGTT-3'	Sense primer for RT-PCR analysis *orf3*	531
**3rtREV**	5'-AGCGCGAGGAACACGACGAGC-3'	Antisense primer for RT-PCR analysis *orf3*	
**4rtFOR**	5'-AACATCCTGCGCTGAAGGTCAAC-3'	Sense primer for RT-PCR analysis *orf4*	537
**4rtREV**	5'-TGACAGCCCCTCTTCCGCCATCAC-3'	Antisense primer for RT-PCR analysis *orf4*	
**5rtFOR**	5'-TACGTGCTGCGCGAACTGAAG-3'	Sense primer for RT-PCR analysis *orf5*	594
**5rtREV**	5'-AACAGCCACATCACGCAAAACAC-3'	Antisense primer for RT-PCR analysis *orf5*	
**6rtFOR**	5'-TCGAATGGGTGCTGAACCGC-3'	Sense primer for RT-PCR analysis *orf6*	477
**6rtREV**	5'-CGAATTCTTCCGAGTTGGGGCT-3'	Antisense primer for RT-PCR analysis *orf6*	
**8rtFOR**	5'-CAAGGTCGTGACCGGCGTGTA-3'	Sense primer for RT-PCR analysis *orf8*	517
**8rtREV**	5'-GATCATCGACAGCACCATCAGC-3'	Antisense primer for RT-PCR analysis *orf8*	
**9rtFOR**	5'-CTATTCGATCAATACGCTCACG-3'	Sense primer for RT-PCR analysis *orf9*	493
**9rtREV**	5'-CGCATAGACGGCGAACACCAC-3'	Antisense primer for RT-PCR analysis *orf9*	
**10rtFOR**	5'-AACTCGCTGCAGATCTCGGAC-3'	Sense primer for RT-PCR analysis *orf10*	521
**10rtREV**	5'-ATCGCGAACTGCTTGTAGAACTG-3'	Antisense primer for RT-PCR analysis *orf10*	
**11rtFOR**	5'-TCGCGGCGAAGATGGAGGAC-3'	Sense primer for RT-PCR analysis *orf11*	522
**11rtREV**	5'-TCGTCGCCATCGGATGGAACAT-3'	Antisense primer for RT-PCR analysis *orf11*	
**12rtFOR**	5'-CGCGCTCAACGACTGGTTCCT-3'	Sense primer for RT-PCR analysis *orf12*	520
**12rtREV**	5'-CAGTTGCGATCGGTCGTAGGTC-3'	Antisense primer for RT-PCR analysis *orf12*	
**13rtFOR**	5'-CAGGTTACACAGATGCGAACGA-3'	Sense primer for RT-PCR analysis *orf13*	530
**13rtREV**	5'-AAGCCCGACACGTGACGAGCG-3'	Antisense primer for RT-PCR analysis *orf13*	
**14rtFOR**	5'-ACGCCGACCGACATCTTTCCG-3'	Sense primer for RT-PCR analysis *orf14*	615
**14rtREV**	5'-CTGCGTGCTGCCGTTCATCTGC-3'	Antisense primer for RT-PCR analysis *orf14*	
**15rtFOR**	5'-AGAGTTTGATCATGGCTCAG-3'	Sense primer for RT-PCR analysis *rRNA *16S gene	1490
**16rtREV**	5'-TACCTTGTTACGACTTCAC-3'	Antisense primer for RT-PCR analysis *rRNA *16S gene	

## References

[B1] Nikaido H (2001). Preventing drug access to targets: cell surface permeability barriers and active efflux in bacteria. Semin Cell Dev Biol.

[B2] Ryan BM, Dougherty TJ, Beaulieu D, Chuang J, Dougherty BA, Barrett JF (2001). Efflux in bacteria: what do we really know about it?. Expert Opin Investig Drugs.

[B3] Li XZ, Nikaido H, Poole K (1995). Role of *mexA-mexB-oprM *in antibiotic efflux in *Pseudomonas aeruginosa*. Antimicrob Agents Chemother.

[B4] Li XZ, Nikaido H (2004). Efflux-mediated drug resistance in bacteria. Drugs.

[B5] Paulsen IT, Brown MH, Skurray RA (1996). Proton-dependent multidrug efflux systems. Microbiol Rev.

[B6] Akama H, Matsuura T, Kashiwagi S, Yoneyama H, Narita S, Tsukihara T, Nakagawa A, Nakae T (2004). Crystal structure of the membrane fusion protein, MexA, of the multidrug transporter in *Pseudomonas aeruginosa*. J Biol Chem.

[B7] Akama H, Kanemaki M, Yoshimura M, Tsukihara T, Kashiwagi T, Yoneyama H, Narita S, Nakagawa A, Nakae T (2004). Crystal structure of the drug discharge outer membrane protein, OprM, of *Pseudomonas aeruginosa*: dual modes of membrane anchoring and occluded cavity end. J Biol Chem.

[B8] Higgins MK, Bokma E, Koronakis E, Hughes C, Koronakis V (2004). Structure of the periplasmic component of a bacterial drug efflux pump. Proc Natl Acad Sci USA.

[B9] Koronakis V, Sharff A, Koronakis E, Luisi B, Hughes C (2000). Crystal structure of the bacterial membrane protein TolC central to multidrug efflux and protein export. Nature.

[B10] Murakami S, Nakashima R, Yamashita E, Yamaguchi A (2002). Crystal structure of bacterial multidrug efflux transporter AcrB. Nature.

[B11] Koronakis V, Eswaran J, Hughes C (2004). Structure and function of TolC: the bacterial exit duct for proteins and drugs. Annu Rev Biochem.

[B12] Aires JR, Nikaido H (2005). Aminoglycosides are captured from both periplasm and cytoplasm by the AcrD multidrug efflux transporter of *Escherichia coli*. J Bacteriol.

[B13] Eswaran J, Koronakis E, Higgins MK, Hughes C, Koronakis V (2004). Three's company: component structures bring a closer view of tripartite drug efflux pumps. Curr Opin Struct Biol.

[B14] Ma D, Cook DN, Alberti M, Pon NG, Nikaido HM, Hearst JE (1993). Molecular cloning of *acrA *and *acrE *genes of *Escherichia coli*. J Bacteriol.

[B15] Aires JR, Kohler T, Nikaido H, Plesiat P (1999). Involvement of an active efflux system in the natural resistance of *Pseudomonas aeruginosa *to aminoglycosides. Antimicrob Agents Chemother.

[B16] Poole K (2001). Multidrug efflux pumps and antimicrobial resistance in *Pseudomonas aeruginosa *and related organisms. J Mol Microbiol Biotechnol.

[B17] Speert DP (2001). Understanding *Burkholderia cepacia*: epidemiology, genomovars, and virulence. Infect Med.

[B18] Lyczak JB, Cannon CL, Pier GB (2002). Lung infections associated with cystic fibrosis. Clin Microbiol Rev.

[B19] Coenye T, Vandamme P, Govan JRW, LiPuma JJ (2001). Taxonomy and identification of the *Burkholderia cepacia *complex. J Clin Microbiol.

[B20] Mahenthiralingam E, Urban TA, Goldberg JB (2005). The multifarious, multireplicon *Burkholderia cepacia *complex. Nat Rev Microbiol.

[B21] Reik R, Spilker T, LiPuma JJ (2005). Distribution of *Burkholderia cepacia *complex species among isolates recovered from persons with or without cystic fibrosis. J Clin Microbiol.

[B22] Quinn JP (1998). Clinical problems posed by multiresistant nonfermenting gram-negative pathogens. Clin Infect Dis.

[B23] Aronoff SC (1988). Outer membrane permeability in *Pseudomonas cepacia*: diminished porin content in a beta-lactam-resistant mutant and in resistant cystic fibrosis isolates. Antimicrob Agents Chemother.

[B24] Moore RA, Hancock RE (1986). Involvement of outer membrane of *Pseudomonas cepacia *in aminoglycoside and polymyxin resistance. Antimicrob Agents Chemother.

[B25] Parr TR, Moore RA, Moore LV, Hancock RE (1987). Role of porins in intrinsic antibiotic resistance of *Pseudomonas cepacia*. Antimicrob Agents Chemother.

[B26] Trepanier S, Prince A, Huletsky A (1997). Characterization of the *penA *and *penR *genes of *Burkholderia cepacia *249 which encode the chromosomal class A penicillinase and its LysR-type transcriptional regulator. Antimicrob Agents Chemother.

[B27] Burns JL, Lien DM, Hedin LA (1989). Isolation and characterization of dihydrofolate reductase from trimethoprim-susceptible and trimethoprim-resistant *Pseudomonas cepacia*. Antimicrob Agents Chemother.

[B28] Fehlner-Gardiner CC, Valvano MA (2002). Cloning and characterization of the *Burkholderia vietnamiensis norM *gene encoding a multi-drug efflux protein. FEMS Microbiol Lett.

[B29] Wigfield SM, Rigg GP, Kavari M, Webb AK, Matthews RC, Burnie JP (2002). Identification of an immunodominant drug efflux pump in *Burkholderia cepacia*. J Antimicrob Chemother.

[B30] Burns JL, Wadsworth CD, Barry JJ, Goodall CP (1996). Nucleotide sequence analysis of a gene from *Burkholderia *(*Pseudomonas*) *cepacia *encoding an outer membrane lipoprotein involved in multiple antibiotic resistance. Antimicrob Agents Chemother.

[B31] Nair BM, Cheung KJ, Griffith A, Burns JL (2004). Salicylate induces an antibiotic efflux pump in *Burkholderia cepacia *complex genomovar III (*B. cenocepacia*). J Clin Invest.

[B32] Govan JR, Brown PH, Maddison J, Doherty CJ, Nelson JW, Dodd M, Greening AP, Webb AK (1993). Evidence for transmission of *Pseudomonas cepacia *by social contact in cystic fibrosis. Lancet.

[B33] The Sanger Centre. http://www.sanger.ac.uk.

[B34] Putman M, van Veen HW, Konings WN (2000). Molecular properties of bacterial multidrug transporters. Microbiol Mol Biol Rev.

[B35] Ma D, Cook DN, Alberti M, Pon NG, Nikaido H, Hearst JE (1995). Genes *acrA *and *acrB *encode a stress-induced efflux system of *Escherichia coli*. Mol Microbiol.

[B36] Hassan MT, van der Lelie D, Springael D, Romling U, Ahmed N, Mergeay M (1999). Identification of a gene cluster, *czr*, involved in cadmium and zinc resistance in *Pseudomonas aeruginosa*. Gene.

[B37] Zgurskaya HI, Nikaido H (2000). Multidrug resistance mechanisms: drug efflux across two membranes. Mol Microbiol.

[B38] Nakamura H (1979). Novel acriflavin resistance genes, *acrC *and *acrD*, in *Escherichia coli *K-12. J Bacteriol.

[B39] Schweizer HP (2003). Efflux as a mechanism of resistance to antimicrobials in *Pseudomonas aeruginosa *and related bacteria: unanswered questions. Genet Mol Res.

[B40] Kumar A, Schweizer HP (2005). Bacterial resistance to antibiotics: active efflux and reduced uptake. Adv Drug Deliv Rev.

[B41] Adewoye L, Sutherland A, Srikumar R, Poole K (2002). The MexR repressor of the *mexAB-oprM *multidrug efflux operon in *Pseudomonas aeruginosa*: characterization of mutations compromising activity. J Bacteriol.

[B42] Wang H, Noordewier M, Benham CJ (2004). Stress-induced DNA duplex destabilization (SIDD) in the *E. coli *genome: SIDD sites are closely associated with promoters. Genome Res.

[B43] Masuda N, Sakagawa E, Ohya S, Gotoh N, Tsujimoto H, Nishino T (2000). Contribution of the MexX-MexY-OprM efflux system to intrinsic resistance in *Pseudomonas aeruginosa*. Antimicrob Agents Chemother.

[B44] Jeannot K, Sobel ML, El Garch F, Poole K, Plesiat P (2005). Induction of the MexXY efflux pump in *Pseudomonas aeruginosa *is dependent on drug-ribosome interaction. J Bacteriol.

[B45] Vogne C, Aires JR, Bailly C, Hocquet D, Plésiat P (2004). Role of the multidrug efflux system MexXY in the emergence of moderate resistance to aminoglycosides among *Pseudomonas aeruginosa *isolates from patients with cystic fibrosis. Antimicrob Agents Chemother.

[B46] Aeschlimann JR (2003). The role of multidrug efflux pumps in the antibiotic resistance of *Pseudomonas aeruginosa *and other gram-negative bacteria. Insights from the Society of Infectious Diseases Pharmacists. Pharmacotherapy.

[B47] Piddock LJV (2006). Clinically relevant chromosomally encoded multidrug resistance efflux pumps in bacteria. Clin Microbiol Rev.

[B48] Morita Y, Kodama K, Shiota S, Mine T, Kataoka A, Mizushima T, Tsuchiya T (1998). NorM, a putative multidrug efflux protein, of *Vibrio parahaemolyticus *and its homolog in *Escherichia coli*. Antimicrob Agents Chemother.

[B49] Masuda N, Ohya S (1992). Cross-resistance to meropenem, cephems, and quinolones in *Pseudomonas aeruginosa*. Antimicrob Agents Chemother.

[B50] Yoneda K, Chikumi H, Murata T, Gotoh N, Yamamoto H, Fujiwara H, Nishino T, Shimizu E (2005). Measurement of *Pseudomonas aeruginosa *multidrug efflux pumps by quantitative real-time polymerase chain reaction. FEMS Microbial Lett.

[B51] Morita Y, Murata T, Mima T, Shiota S, Kuroda T, Mizushima T, Gotoh N, Nishino T, Tsuchiya T (2003). Induction of *mexCD-oprJ *operon for a multidrug efflux pump by disinfectants in wild-type *Pseudomonas aeruginosa *PAO1. J Antimicrob Chemother.

[B52] Lomovskaya O, Watkins WJ (2001). Efflux pumps: their role in antibacterial drug discovery. Curr Med Chem.

[B53] The Sanger FTP Server. ftp://ftp.sanger.ac.uk/pub/pathogens/bc/.

[B54] The European Bioinformatics Institute. http://www.ebi.ac.uk/.

[B55] National Centre for Biotechnology Information. http://www.ncbi.nlm.nih.gov/.

[B56] Center for Biological Sequence Analysis. http://www.cbs.dtu.dk/.

[B57] Structural Bioinformatics Group. http://www.sbg.bio.ic.ac.uk/.

[B58] Genome Center. http://www.genomecenter.ucdavis.edu/.

[B59] Benham CJ (1993). Sites of predicted stress-induced DNA duplex destabilization occur preferentially at regulatory loci. Proc Natl Acad Sci USA.

[B60] Scordilis GE, Ree H, Lessie TG (1987). Identification of transposable elements which activate gene expression in *Pseudomonas cepacia*. J Bacteriol.

[B61] Sambrook J, Russell DW (2001). *Molecular cloning: a laboratory manual*.

